# The intra- and inter-day repeatability of corneal densitometry measurements in subjects with keratoconus and in healthy controls

**DOI:** 10.1038/s41598-023-32822-y

**Published:** 2023-04-05

**Authors:** Ingemar Gustafsson, Dimitrios Bizios, Anders Ivarsen, Jesper Ø. Hjortdal

**Affiliations:** 1 Department of Clinical Sciences, Ophthalmology, Lund University, Lund, Sweden; 2 Department of Ophthalmology, Aarhus University Hospital, Aarhus, Denmark; 3 Department of Ophthalmology, Skåne University Hospital, Kioskgatan 1 , 221 85 Lund, Sweden

**Keywords:** Health care, Eye diseases

## Abstract

The healthy cornea is transparent, however, disease can affect its structure, rendering it more or less opaque. The ability to assess the clarity of the cornea objectively could thus be of considerable interest for keratoconus patients. It has previously been suggested that densitometry can be used to diagnose early keratoconus, and that the values of densitometry variables increase with increasing disease severity, indicating that densitometry could also be used to assess progressive keratoconus. Previous studies have only assessed the repeatability of corneal densitometry measurements on the same day, which does not reflect the clinical setting in which changes are evaluated over time. We have therefore evaluated the inter-day repeatability of densitometry measurements in both patients with keratoconus and healthy controls. Measurements in the middle layer of the 2–6 mm zone of the cornea showed the best repeatability. Although an objective measure of the corneal transparency could be interesting, the generally poor repeatability of densitometry measurements limits their use. The repeatability of corneal clarity measurements could be improved by using other approaches such as optical coherence tomography, but this remains to be investigated. Such improvements would allow the more widespread use of corneal densitometry in clinical practice.

## Introduction

The cornea is transparent due to its unique arrangement of collagen fibrillae in lamellar layers^1^. The direction of the fibrillae in each lamella is perpendicular to the direction of those in the next layer. This structure, together with the size of the fibrillae and the size of the inter-fibrillar spacing causes destructive interference of scattered light affording the cornea its transparency^2,3^. However, if the corneal structure is disrupted, the back scattering of incoming light will increase, reducing its transparency.

**Figure 1:**
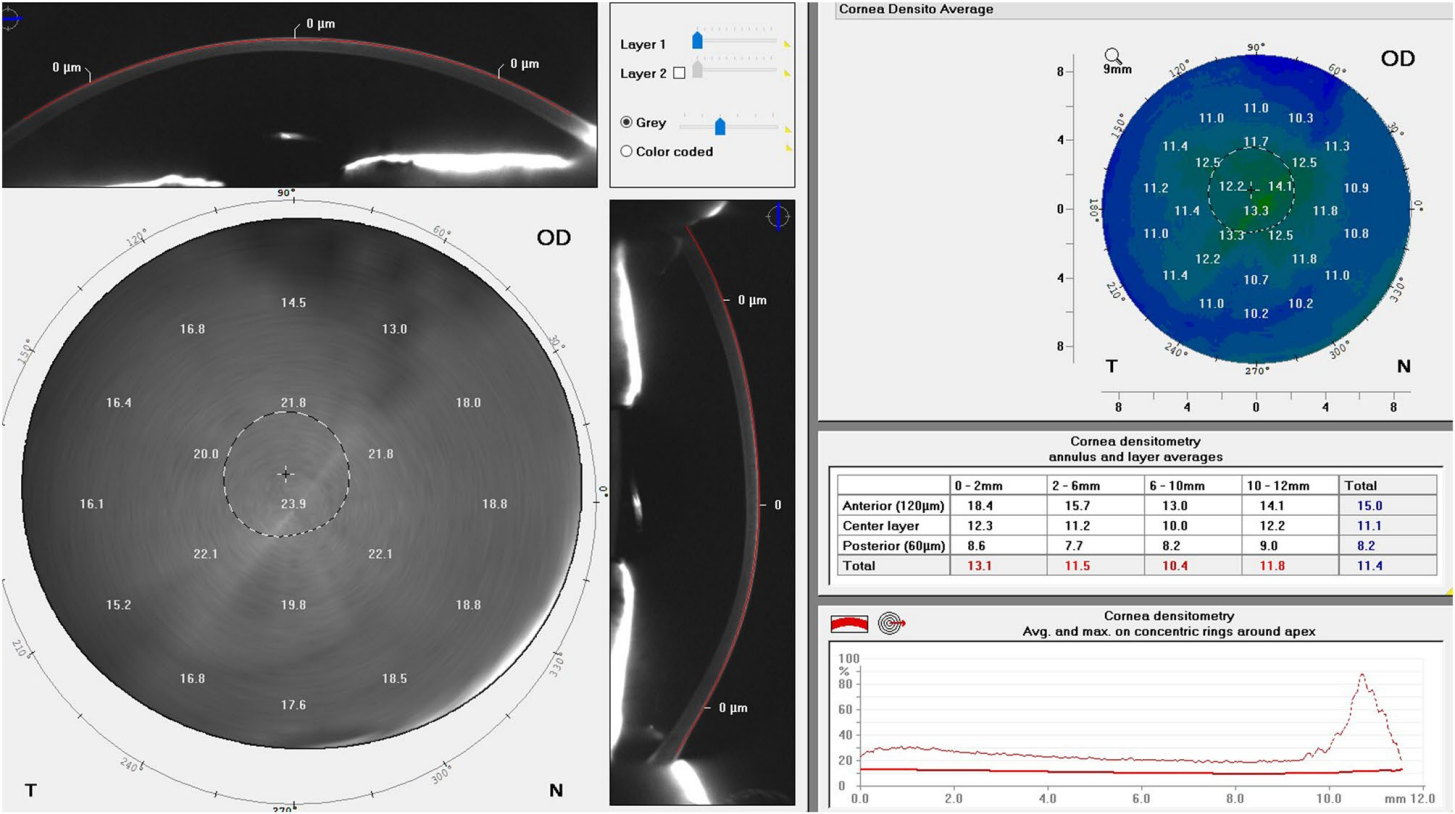
Image of the densitomtry module in the Pentacam HR.

Several techniques have been suggested for the measurement of backscattering in the cornea, for example, optical coherence tomography (OCT)^4^, confocal microscopy^5^ and Scheimpflug imaging^6^. However, the Pentacam HR Scheimpflug camera is the only system commercially available for assessing corneal transparency^7^. The Pentacam HR measures the backscattered light to provide a value of the corneal transparency^7^. The value is presented in greyscale units, where 0 indicates completely transparent and 100 completely opaque (Fig. 1). Many corneal pathologies can reduce the corneal transparency, which is commonly evaluated by a subjective assessment of visual acuity. However, an objective measure would be of considerable value.

Keratoconus is a corneal disease that causes a reduction in visual acuity and distorted vision. Keratoconus usually manifests within the first two decades of life, and progression of the disease can lead to severe deterioration in vision^8^. Progressive keratoconus can be halted by corneal crosslinking (CXL)^9^, and it is therefore of paramount importance to be able to detect progression accurately^10^. Changes in visual acuity are often used to assess progression^11^, as well as various tomographic parameters^12^. As keratoconus is a degenerative disorder of the corneal stroma, the reduction in biomechanical strength causes the cornea to protrude^13^. This leads to derangement of the corneal collagen fibrillae and consequently an increase in corneal backscattering^2^. In fact, it has been suggested that corneal densitometry can be used both to detect keratoconus^14^, and possibly to assess progression as the magnitude of the densitometry has been suggested to correlate with the severity of keratoconus^15^.

A few studies have been carried out on the intra-day repeatability of densitometry in subjects with keratoconus^16,17^, and in healthy subjects^6,18^. However, as clinical measurements are compared over time, the inter-day repeatability is more relevant. No such publications could be found in the literature, and the purpose of this study was therefore to analyse the inter-day repeatability of corneal densitometry measurements in subjects with keratoconus and in healthy controls.

## Results

**Figure 2:**
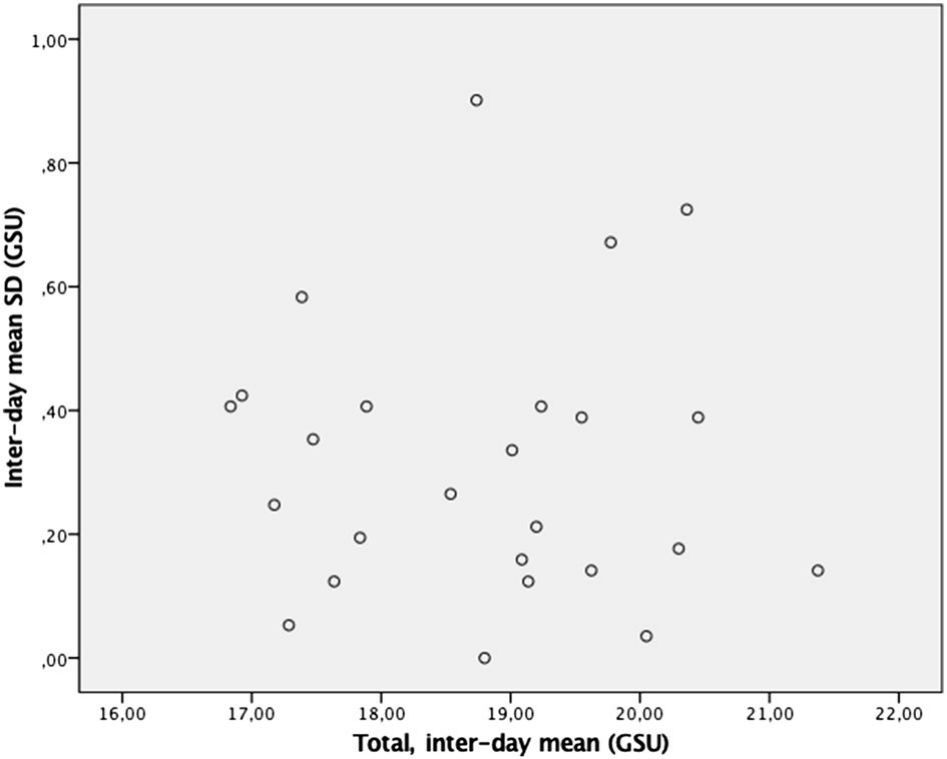
Mean standard deviation in the total densitometry plotted against the mean interday values of total densitometry for the keratoconus subjects.

The standard deviation of the measurements was plotted against the mean value to evaluate any possible association between increasing standard deviation and increasing value of the parameter. Some associations were found, for example, a statistically significant negative association was found in the anterior layer in the 2–6 mm zone (*p* = 0.005) among healthy controls (SI 1), indicating better repeatability in subjects with less transparent corneae. A statistically significant negative association was also found for the total value in the 2–6 mm zone (*p* = 0.038) among subjects with keratoconus (SI 2). Furthermore, a statistically significant association, showing poorer repeatability in subjects with less transparent corneas (*p* = 0.025), was found in the posterior layer in the 10–12 mm zone in subjects with keratoconus (SI 3). However, these associations are more likely to be explained by a generally high variability of the measurements between days, rather than a significant association per se. Indeed, the high variability of the measurements appears to be similar regardless of the densitometry value (Fig. 2). Inspection of the data suggested that these associations could be explained by a few outliers.

### The intra-day repeatability of measurements in subjects with keratoconus and healthy controls

Descriptive statistics and the repeatability of the measurements are presented in Table 1 (keratoconus cohort) and Table 2 (healthy controls). The ICC was higher in the healthy cohort than in the keratoconus cohort. A higher ICC suggests that the variability between measurements is more likely to be due to inter-subject differences than intra-subject variability, thus suggesting a higher variability of the measurements among subjects with keratoconus. The repeatability was indeed better in healthy controls for all annular segments (0–2, 2–6, 6–10 and 10–12 mm), and at all depths (anterior, middle and posterior layers).

**Table 1:** Descriptive statistics and repeatability of measurements on Day 0 and Day 3 in subjects with keratoconus.

Variable	Day	Mean (SD)^a^	Min‒Max	S_w_ (95% CI)	CV%	Repeatability	ICC (95% CI)
0‒2 mm							
Anterior	0	27.9 (2.2)	(23.4‒33.0)	0.73 (0.62‒0.85)	2.62	2.04	0.90 (0.82‒0.95)
	3	28.2 (2.1)	(23.8‒32.7)	0.62 (0.52‒0.72)	2.20	1.73	0.92 (0.86‒0.96)
Middle	0	17.1 (1.2)	(14.9‒19.3)	0.44 (0.37‒0.50)	2.57	1.21	0.88 (0.80‒0.94)
	3	17.1 (1.0)	(15.2‒19.1)	0.43 (0.36‒0.50)	2.51	1.18	0.85 (0.75‒0.92)
Posterior	0	12.8 (0.9)	(10.8‒15.2)	0.69 (0.58‒0.80)	5.40	1.92	0.62 (0.44‒0.78)
	3	12.7 (0.9)	(10.9‒14.8)	0.63 (0.53‒0.73)	4.97	1.73	0.64 (0.46‒0.80)
Total	0	19.2 (1.3)	(16.7‒22.0)	0.54 (0.45‒0.62)	2.81	1.48	0.84 (0.73‒0.92)
	3	19.4 (1.2)	(16.9‒21.8)	0.45 (0.38‒0.52)	2.32	1.24	0.87 (0.78‒0.93)
2‒6 mm							
Anterior	0	24.3 (1.7)	(20.8‒28.6)	0.46 (0.39‒0.53)	1.89	1.27	0.93 (0.88‒0.97)
	3	24.5 (1.6)	(20.7‒28.2)	0.41 (0.35‒0.48)	1.68	1.15	0.94 (0.89‒0.97)
Middle	0	15.0 (0.9)	(13.4‒16.8)	0.34 (0.28‒0.39)	2.27	0.93	0.87 (0.78‒0.93)
	3	15.0 (0.8)	(13.3‒16.5)	0.23 (0.19‒0.26)	1.53	0.63	0.92 (0.87‒0.96)
Posterior	0	12.2 (0.9)	(10.8‒14.5)	0.46 (0.39‒0.53)	3.78	1.27	0.77 (0.63‒0.88)
	3	12.2 (0.8)	(10.9‒14.5)	0.39 (0.33‒0.46)	3.20	1.09	0.82 (0.70‒0.90)
Total	0	17.2 (1.1)	(15.1‒20.0)	0.36 (0.30‒0.42)	2.09	1.00	0.89 (0.82‒0.95)
	3	17.2 (1.0)	(14.9‒19.7)	0.28 (0.24‒0.33)	1.63	0.79	0.92 (0.87‒0.96)
6‒10 mm							
Anterior	0	22.6 (3.1)	(14.7‒29.1)	2.23 (1.87‒2.59)	9.87	6.18	0.62 (0.44‒0.79)
	3	22.6 (2.5)	(18.2‒27.6)	0.69 (0.58‒0.81)	3.05	1.93	0.93 (0.87‒0.96)
Middle	0	15.4 (1.9)	(12.3‒19.6)	0.69 (0.58‒0.80)	4.48	1.90	0.89 (0.80‒0.94)
	3	15.2 (1.8)	(12.1‒19.2)	0.35 (0.30‒0.41)	2.30	0.98	0.96 (0.94‒0.98)
Posterior	0	13.9 (2.1)	(10.8‒19.6)	0.60 (0.50‒0.70)	4.32	1.66	0.92 (0.86‒0.96)
	3	13.8 (1.9)	(10.8‒19.5)	0.40 (0.34‒0.47)	2.90	1.12	0.96 (0.92‒0.98)
Total	0	17.3 (2.1)	(13.8‒22.2)	0.83 (0.70‒0.97)	4.80	2.31	0.86 (0.76‒0.93)
	3	17.2 (1.9)	(14.6‒21.1)	0.57 (0.48‒0.66)	3.31	1.57	0.92 (0.85‒0.96)
10‒12 mm							
Anterior	0	32.7 (7.5)	(22.2‒50.9)	2.83 (2.38‒3.28)	8.65	7.84	0.87 (0.78‒0.93)
	3	32.1 (5.9)	(23.9‒47.2)	5.43 (4.56‒6.30)	16.92	15.06	0.48 (0.28‒0.68)
Middle	0	21.9 (3.6)	(15.5‒29.4)	1.30 (1.09‒1.50)	5.93	3.59	0.88 (0.80‒0.94)
	3	22.1 (3.4)	(16.8‒28.8)	1.56 (1.31‒1.81)	7.06	4.32	0.82 (0.70‒0.91)
Posterior	0	21.9 (3.6)	(13.4‒25.3)	1.09 (0.92‒1.27)	4.98	3.03	0.91 (0.85‒0.96)
	3	19.6 (3.4)	(13.3‒26.7)	1.08 (0.91‒1.25)	5.51	2.99	0.91 (0.84‒0.95)
Total	0	24.3 (4.4)	(18.0‒34.8)	2.32 (1.94‒2.69)	9.55	6.42	0.77 (0.63‒0.88)
	3	24.6 (3.9)	(19.2‒33.7)	2.35 (1.98‒2.73)	9.56	6.52	0.71 (0.55‒0.84)
Total							
Anterior	0	25.7 (1.9)	(22.8‒30.5)	0.69 (0.58‒0.80)	2.68	1.90	0.88 (0.80‒0.94)
	3	25.7 (2.0)	(22.3‒30.0)	0.87 (0.73‒1.01)	3.39	2.42	0.83 (0.72‒0.91)
Middle	0	16.6 (1.2)	(14.1‒19.1)	0.62 (0.52‒0.72)	3.73	1.72	0.78 (0.64‒0.88)
	3	16.6 (1.1)	(14.7‒18.6)	0.35 (0.30‒0.41)	2.11	0.97	0.90 (0.83‒0.95)
Posterior	0	14.1 (1.4)	(12.1‒17.1)	1.04 (0.87‒1.21)	7.38	2.88	0.60 (0.42‒0.77)
	3	13.9 (1.0)	(12.4‒15.6)	1.09 (0.92‒1.27)	7.84	3.03	0.35 (0.15‒0.57)
Total	0	18.8 (1.3)	(16.6‒21.5)	0.61 (0.52‒0.71)	3.24	1.70	0.81 (0.69‒0.90)
	3	18.7 (1.3)	(17.0‒21.3)	0.49 (0.41‒0.57)	2.62	1.37	0.86 (0.76‒0.93)

^a^Subject mean. The values are presented in greyscale units. Standard deviation (SD), S_w_ (Within-subject standard deviation, CI (confidence intervals), CV% (coefficient of variation), ICC (Intraclass correlation coefficient).

**Table 2:** Descriptive statistics and repeatability of measurements on Day 0 and Day 3 in healthy controls.

Variable	Day	Mean (SD)^a^	Min‒Max	Sw (95% CI)	CV%	Repeatability	ICC (95% CI)
0‒2 mm							
Anterior	0	22.6 (4.4)	(16.1‒29.4)	0.40 (0.34‒0.46)	1.78	1.11	0.99 (0.98‒1.00)
	3	23.0 (4.1)	(17.3‒29.3)	0.29 (0.25‒0.34)	1.26	0.82	0.99 (0.99‒1.00)
Middle	0	14.3 (2.5)	(10.6‒18.3)	0.25 (0.21‒0.29)	1.75	0.70	0.99 (0.98‒0.99)
	3	14.4 (2.3)	(11.7‒18.0)	0.24 (0.21‒0.28)	1.67	0.68	0.99 (0.98‒0.99)
Posterior	0	11.8 (1.4)	(9.4‒13.9)	0.39 (0.33‒0.46)	3.30	1.09	0.93 (0.87‒0.96)
	3	11.9 (1.3)	(10.1‒14.1)	0.34 (0.29‒0.39)	2.86	0.94	0.93 (0.89‒0.97)
Total	0	16.2 (2.7)	(11.9‒20.5)	0.24 (0.21‒0.28)	1.48	0.68	0.99 (0.99‒1.00)
	3	16.4 (2.5)	(13.1‒20.4)	0.23 (0.20‒0.27)	1.40	0.64	0.99 (0.98‒1.00)
2‒6 mm							
Anterior	0	20.3 (3.9)	(14.5‒25.9)	0.20 (0.17‒0.23)	0.99	0.55	1.00 (1.00‒1.00)
	3	20.7 (3.6)	(15.9‒25.7)	0.48 (0.40‒0.55)	2.32	1.32	0.98 (0.97‒0.99)
Middle	0	12.9 (2.1)	(9.9‒15.8)	0.13 (0.11‒0.16)	1.01	0.37	1.00 (0.99‒1.00)
	3	13.1 (2.0)	(10.8‒15.7)	0.32 (0.27‒0.37)	2.44	0.89	0.97 (0.95‒0.99)
Posterior	0	11.0 (1.3)	(9.0‒13.7)	0.30 (0.26‒0.35)	2.73	0.84	0.95 (0.91‒0.97)
	3	11.1 (1.2)	(9.6‒14.0)	0.39 (0.33‒0.46)	3.51	1.09	0.91 (0.84‒0.95)
Total	0	14.8 (2.4)	(11.2‒18.1)	0.17 (0.14‒0.19)	1.15	0.46	1.00 (0.99‒1.00)
	3	15.0 (2.2)	(12.2‒18.1)	0.36 (0.30‒0.42)	2.40	1.00	0.97 (0.95‒0.99)
6‒10 mm							
Anterior	0	18.9 (3.9)	(13.6‒28.6)	1.01 (0.85‒1.17)	5.35	2.79	0.94 (0.89‒0.97)
	3	19.1 (3.9)	(14.2‒29.9)	0.52 (0.43‒0.60)	2.72	1.43	0.98 (0.97‒0.99)
Middle	0	13.2 (2.5)	(9.4‒19.7)	0.41 (0.35‒0.48)	3.11	1.15	0.97 (0.95‒0.99)
	3	13.3 (2.5)	(10.3‒20.8)	0.29 (0.24‒0.34)	2.18	0.81	0.99 (0.98‒0.99)
Posterior	0	12.3 (2.1)	(9.0‒17.7)	0.31 (0.26‒0.36)	2.52	0.87	0.98 (0.96‒0.99)
	3	12.4 (2.1)	(9.9‒18.6)	0.28 (0.23‒0.32)	2.26	0.77	0.98 (0.97‒0.99)
Total	0	14.8 (2.8)	(10.7‒22.0)	0.56 (0.47‒0.64)	3.78	1.54	0.96 (0.93‒0.98)
	3	14.9 (2.8)	(11.6‒23.1)	0.33 (0.28‒0.38)	2.21	0.92	0.99 (0.97‒0.99)
10‒12 mm							
Anterior	0	28.0 (7.7)	(15.2‒49.3)	1.65 (1.38‒1.91)	5.89	4.57	0.96 (0.92‒0.98)
	3	27.2 (7.9)	(17.8‒47.0)	1.49 (1.25‒1.73)	5.48	4.13	0.97 (0.94‒0.98)
Middle	0	18.8 (3.9)	(12.3‒28.4)	0.70 (0.59‒0.82)	3.72	1.95	0.97 (0.94‒0.98)
	3	18.4 (4.0)	(13.1‒28.0)	0.65 (0.55‒0.76)	3.53	1.81	0.97 (0.95‒0.99)
Posterior	0	16.1 (3.6)	(10.6‒25.4)	0.57 (0.48‒0.66)	3.54	1.58	0.98 (0.96‒0.99)
	3	16.0 (3.6)	(10.9‒25.5)	0.59 (0.49‒0.68)	3.69	1.63	0.97 (0.95‒0.99)
Total	0	21.0 (4.6)	(12.7‒33.9)	0.91 (0.76‒1.05)	4.33	2.52	0.96 (0.93‒0.98)
	3	20.4 (4.7)	(14.5‒33.0)	1.31 (1.10‒1.51)	6.42	3.62	0.93 (0.87‒0.96)
Total							
Anterior	0	21.5 (4.1)	(15.4‒30.0)	0.34 (0.28‒0.39)	1.58	0.93	0.99 (0.99‒1.00)
	3	21.6 (3.9)	(16.4‒30.2)	0.37 (0.31‒0.43)	1.71	1.02	0.99 (0.98‒1.00)
Middle	0	14.2 (2.4)	(10.4‒18.9)	0.18 (0.15‒0.20)	1.27	0.49	0.99 (0.99‒1.00)
	3	14.4 (2.4)	(11.3‒19.4)	0.78 (0.66‒0.91)	5.42	2.17	0.90 (0.84‒0.95)
Posterior	0	12.4 (1.9)	(9.4‒16.4)	0.30 (0.26‒0.35)	2.42	0.84	0.97 (0.95‒0.99)
	3	12.6 (1.9)	(10.3‒17.0)	0.85 (0.71‒0.98)	6.74	2.35	0.83 (0.72‒0.91)
Total	0	16.0 (2.7)	(11.7‒21.8)	0.21 (0.17‒0.24)	1.31	0.58	0.99 (0.99‒1.00)
	3	16.2 (2.7)	(12.9‒22.2)	0.74 (0.62‒0.85)	4.57	2.04	0.93 (0.88‒0.96)

^a^Subject mean. The values are presented in greyscale units. Standard deviation (SD), S_w_ (Within-subject standard deviation, CI (confidence intervals), CV% (coefficient of variation), ICC (Intraclass correlation coefficient).

A feature common to both cohorts was that the 2–6 mm zone showed the best repeatability, followed by the 0–2 mm zone. The outer zones, 6–10 and 10–12 mm, showed poorer repeatability. In terms of corneal depth, both cohorts showed better repeatability in the middle layer, followed by the posterior layer; the poorest repeatability being observed in the anterior layer.

### The inter-day repeatability of single measurements in subjects with keratoconus and in healthy controls

Prediction limits for differences between single measurements are presented in Table 3 (keratoconus) and Table 4 (healthy controls). The inter-day repeatability is presented as *prediction limits* as these describe the predicted value for single measurements. The prediction limits were narrower (best) in the 2–6 mm zone than in the other zones in both cohorts. In terms of prediction limits at different depths in the cornea, the middle layer generally showed the narrowest (best) prediction limits, followed by the posterior layer and the anterior layer, which showed the widest (poorest) prediction limits. These findings were similar for both cohorts.

**Table 3:** Prediction limits for differences between single measurements in subjects with keratoconus.

Variable	${\widehat{\tau }}^{2}$	${\widehat{\sigma }}_{1}^{2}$	${\widehat{\sigma }}_{2}^{2}$	${\widehat{\alpha }}_{1}-{\widehat{\alpha }}_{2}$	${\widehat{\alpha }}_{1}-{\widehat{\alpha }}_{2}-2\times \sqrt{2{\widehat{\tau }}^{2}+{\widehat{\sigma }}_{1}^{2}+{\widehat{\sigma }}_{2}^{2}}$	${\widehat{\alpha }}_{1}-{\widehat{\alpha }}_{2}+2\times \sqrt{2{\widehat{\tau }}^{2}+{\widehat{\sigma }}_{1}^{2}+{\widehat{\sigma }}_{2}^{2}}$
Densito 0–2 mm						
Anterior	0.19	0.54	0.39	− 0.31	− 2.59	1.98
Middle	0.05	0.19	0.18	− 0.09	− 1.45	1.27
Posterior	0.01	0.48	0.39	0.07	− 1.81	1.95
Total	0.06	0.29	0.20	− 0.11	− 1.66	1.44
Densito 2–6 mm						
Anterior	0.17	0.21	0.17	− 0.14	− 1.83	− 1.83
Middle	0.05	0.11	0.05	− 0.01	− 1.02	1.01
Posterior	0.01	0.21	0.15	0.02	− 1.23	1.26
Total	0.05	0.13	0.08	− 0.05	− 1.17	1.08
Densito 6–10 mm						
Anterior	0.84	4.97	0.48	0.01	− 5.33	5.36
Middle	0.17	0.47	0.13	0.12	− 1.82	2.07
Posterior	0.14	0.36	0.16	0.14	− 1.65	1.94
Total	0.27	0.70	0.32	0.11	− 2.39	2.61
Densito 10–12 mm						
Anterior	4.44	8.00	29.52	0.57	− 13.05	14.19
Middle	0.87	1.68	2.43	− 0.22	− 5.06	4.61
Posterior	0.26	1.19	1.17	− 0.02	− 3.41	3.37
Total	1.46	5.36	5.53	− 0.31	− 7.74	7.12
Densito Total mm						
Anterior	0.20	0.47	0.76	0.01	− 2.55	2.57
Middle	0.05	0.39	0.12	0.06	− 1.51	1.62
Posterior	0.00	1.07	1.18	0.26	− 2.73	3.26
Total	0.07	0.38	0.24	0.08	− 1.67	1.83

The values are given in greyscale units.

**Table 4:** Prediction limits for differences between single measurements in healthy controls.

Variable	${\widehat{\tau }}^{2}$	${\widehat{\sigma }}_{1}^{2}$	${\widehat{\sigma }}_{2}^{2}$	${\widehat{\alpha }}_{1}-{\widehat{\alpha }}_{2}$	${\widehat{\alpha }}_{1}-{\widehat{\alpha }}_{2}-2\times \sqrt{2{\widehat{\tau }}^{2}+{\widehat{\sigma }}_{1}^{2}+{\widehat{\sigma }}_{2}^{2}}$	${\widehat{\alpha }}_{1}-{\widehat{\alpha }}_{2}+2\times \sqrt{2{\widehat{\tau }}^{2}+{\widehat{\sigma }}_{1}^{2}+{\widehat{\sigma }}_{2}^{2}}$
Densito 0–2 mm						
Anterior	0.27	0.16	0.09	− 0.41	− 2.18	1.35
Middle	0.08	0.06	0.06	− 0.14	− 1.20	0.92
Posterior	0.13	0.16	0.12	− 0.14	− 1.60	1.33
Total	0.14	0.06	0.05	− 0.21	− 1.46	1.03
Densito 2–6 mm						
Anterior	0.20	0.04	0.23	− 0.38	− 2.02	1.25
Middle	0.063	0.02	0.10	− 0.17	− 1.17	0.82
Posterior	0.09	0.09	0.15	− 0.15	− 1.46	1.15
Total^a^	0.10	0.03	0.13	− 0.24	− 1.43	0.95
Densito 6–10 mm						
Anterior	0.29	1.01	0.27	− 0.22	− 2.96	2.51
Middle	0.15	0.17	0.08	− 0.10	− 1.60	1.39
Posterior	0.16	0.10	0.08	− 0.10	− 1.51	1.30
Total	0.18	0.31	0.11	− 0.13	− 1.89	1.62
Densito 10–12 mm						
Anterior	4.10	2.72	2.22	0.86	− 6.39	8.11
Middle	0.95	0.49	0.42	0.36	− 3.00	3.72
Posterior	0.83	0.32	0.34	0.11	− 2.93	3.16
Total	1.42	0.82	1.71	0.52	− 4.11	5.16
Densito Total						
Anterior	0.35	0.11	0.13	− 0.14	− 2.07	1.80
Middle	0.11	0.03	0.61	− 0.14	− 2.01	1.72
Posterior^a^	0.18	0.09	0.72	− 0.16	− 2.31	2.00
Total	0.18	0.04	0.54	− 0.16	− 2.11	1.79

The values are in GSU (Gray Scale Units).

### The inter-day repeatability of the mean of four replicate measurements in subjects with keratoconus and in healthy controls

Descriptive statistics and the repeatability the mean of four replicate measurements in subjects with keratoconus and healthy controls are presented in Table 5. The repeatability of the measurements was best in the middle 0–2 mm zone and in the 2–6 mm zone but deteriorated in the outer zones. In terms of corneal depth, the repeatability was best in the middle layer followed by the posterior layer, and poorest in the anterior layer in subjects with keratoconus and in healthy controls.

**Table 5:** Descriptive statistics and inter-day repeatability of measurements using a mean of four replicates in subjects with keratoconus and healthy controls.

Variable	Group	Mean (SD)^a^	Min‒Max^a^	Sw (95% CI)	CV%	Repeatability	ICC (95% CI)
0‒2 mm							
Anterior	Keratoconus	28.0 (2.1)	(23.6‒32.9)	0.58 (0.42‒0.75)	2.07	1.62	0.93 (0.84‒0.97)
	Control	22.8 (4.2)	(17.1‒29.3)	0.61 (0.44‒0.78)	2.68	1.69	0.98 (0.95‒0.99)
Middle	Keratoconus	17.1 (1.1)	(15.0‒19.1)	0.30 (0.22‒0.39)	1.75	0.84	0.93 (0.84‒0.97)
	Control	14.4 (2.4)	(11.2‒18.2)	0.32 (0.23‒0.40)	2.22	0.88	0.98 (0.96‒0.99)
Posterior	Keratoconus	12.8 (0.9)	(10.9‒15.0)	0.34 (0.24‒0.43)	2.66	0.94	0.87 (0.72‒0.94)
	Control	11.8 (1.3)	(9.9‒13.9)	0.41 (0.30‒0.53)	3.47	1.14	0.91 (0.80‒0.96)
Total	Keratoconus	19.3 (1.2)	(16.8‒21.9)	0.34 (0.25‒0.44)	1.76	0.95	0.92 (0.83‒0.96)
	Control	16.3 (2.6)	(12.6‒20.4)	0.41 (0.30‒0.52)	2.52	1.14	0.98 (0.95‒0.99)
2‒6 mm							
Anterior	Keratoconus	24.4 (1.6)	(20.7‒28.4)	0.46 (0.34‒0.59)	1.89	1.29	0.92 (0.84‒0.97)
	Control	20.5 (3.7)	(15.4‒25.8)	0.55 (0.39‒0.70)	2.68	1.51	0.98 (0.95‒0.99)
Middle	Keratoconus	15.0 (0.8)	(13.3‒16.6)	0.25 (0.18‒0.32)	1.67	0.70	0.91 (0.81‒0.96)
	Control	13.0 (2.0)	(10.4‒15.7)	0.30 (0.22‒0.38)	2.31	0.83	0.98 (0.95‒0.99)
Posterior	Keratoconus	12.2 (0.8)	(11.0‒14.5)	0.23 (0.17‒0.30)	1.89	0.64	0.93 (0.84‒0.97)
	Control	11.1 (1.3)	(9.4‒13.9)	0.36 (0.26‒0.45)	3.24	0.99	0.92 (0.84‒0.97)
Total	Keratoconus	17.2 (1.0)	(15.0‒19.8)	0.28 (0.20‒0.35)	1.63	0.77	0.93 (0.85‒0.97)
	Control	14.9 (2.3)	(11.7‒18.0)	0.38 (0.27‒0.48)	2.55	1.04	0.97 (0.94‒0.99)
6‒10 mm							
Anterior	Keratoconus	22.6 (2.7)	(17.5‒26.6)	1.21 (0.87‒1.54)	5.35	3.35	0.81 (0.63‒0.91)
	Control	19.0 (3.9)	(14.4‒29.2)	0.68 (0.49‒0.87)	3.58	1.88	0.97 (0.93‒0.99)
Middle	Keratoconus	15.3 (1.9)	(12.2‒19.2)	0.50 (0.36‒0.63)	3.27	1.38	0.93 (0.85‒0.97)
	Control	13.3 (2.5)	(9.9‒20.3)	0.43 (0.31‒0.54)	3.23	1.18	0.97 (0.94‒0.99)
Posterior	Keratoconus	13.9 (1.9)	(10.8‒19.6)	0.46 (0.33‒0.58)	3.31	1.27	0.95 (0.88‒0.98)
	Control	12.3 (2.0)	(9.5‒18.2)	0.42 (0.31‒0.54)	3.41	1.17	0.96 (0.91‒0.98)
Total	Keratoconus	17.3 (2.0)	(14.2‒21.0)	0.62 (0.45‒0.80)	3.58	1.73	0.90 (0.80‒0.96)
	Control	14.9 (2.8)	(11.2‒22.6)	0.48 (0.35‒0.61)	3.22	1.33	0.97 (0.93‒0.99)
10‒12 mm							
Anterior	Keratoconus	32.4 (6.4)	(23.1‒46.2)	2.99 (2.16‒3.82)	9.23	8.28	0.80 (0.61‒0.91)
	Control	27.6 (7.6)	(16.5‒48.1)	2.21 (1.60‒2.83)	8.01	6.14	0.92 (0.83‒0.96)
Middle	Keratoconus	22.0 (3.4)	(16.2‒29.1)	1.16 (0.84‒1.49)	5.27	3.23	0.89 (0.77‒0.95)
	Control	18.6 (3.9)	(13.0‒28.2)	1.04 (0.75‒1.33)	5.59	2.89	0.93 (0.85‒0.97)
Posterior	Keratoconus	19.5 (3.5)	(13.3‒25.8)	0.73 (0.53‒0.93)	3.74	2.02	0.96 (0.91‒0.98)
	Control	16.0 (3.5)	(11.3‒25.5)	0.94 (0.68‒1.20)	5.88	2.60	0.93 (0.85‒0.97)
Total	Keratoconus	24.5 (3.9)	(18.6‒34.2)	1.66 (1.20‒2.12)	6.78	4.60	0.84 (0.67‒0.92)
	Control	20.7 (4.5)	(13.6‒33.4)	1.34 (0.97‒1.72)	6.47	3.72	0.92 (0.82‒0.96)
Total							
Anterior	Keratoconus	25.7 (1.9)	(22.8‒30.2)	0.58 (0.42‒0.75)	2.26	1.62	0.91 (0.81‒0.96)
	Control	21.6 (4.0)	(16.2‒30.1)	0.61 (0.44‒0.78)	2.82	1.68	0.98 (0.95‒0.99)
Middle	Keratoconus	16.6 (1.1)	(14.4‒18.5)	0.33 (0.24‒0.43)	1.99	0.92	0.92 (0.82‒0.96)
	Control	14.3 (2.4)	(10.9‒19.1)	0.44 (0.32‒0.57)	3.08	1.23	0.97 (0.93‒0.99)
Posterior	Keratoconus	14.0 (1.1)	(12.4‒16.2)	0.53 (0.38‒0.67)	3.79	1.46	0.81 (0.61‒0.91)
	Control	12.5 (1.8)	(9.9‒16.7)	0.53 (0.38‒0.68)	4.24	1.47	0.92 (0.83‒0.96)
Total	Keratoconus	18.8 (1.3)	(16.8‒21.4)	0.38 (0.28‒0.49)	2.02	1.07	0.91 (0.81‒0.96)
	Control	16.1 (2.7)	(12.3‒22.0)	0.51 (0.37‒0.65)	3.17	1.41	0.96 (0.92‒0.98)

^a^Subject mean. The values are presented in greyscale units. Standard deviation (SD), S_w_ (Within-subject standard deviation, CI (confidence intervals), CV% (coefficient of variation), ICC (Intraclass correlation coefficient).

## Discussion

When assessing the repeatability of measurements, it is necessary to know whether the measurement error is the same for all subjects. If this is not the case, the results will not be representative of the investigated cohort^19,20^. It has been demonstrated in previous studies that the error in the measurement of keratometric parameters increases with increasing disease severity in keratoconus^10,21,22^, and the disease severity must thus be considered. The results of the present study revealed no such correlation in measurements of corneal transparency, in subjects with keratoconus (Stages 1–2) nor in healthy controls. This aspect has not been evaluated or reported previously.

The findings of this study also show that measurements of the transparency of the middle 0–2 and 2–6-mm annular zones have better repeatability than the more outer zones (6–10 and 10–12 mm). The poorer repeatability in the more peripheral zones could be explained by the inclusion of the limbal and sceral area in the analysis, particularly in eyes with diameters less than 12 mm^18^. Another explanation could be that eyelids and lashes have a negative effect on the measurements. Age-related changes in the limbal area such as arcus senilis and crocodile shagreen can increase the densitometry values^6^, however we deemed this to be of little importance as the mean age was 27 years (range 21–45 years) in the keratoconus cohort and 29 years (range 23–41 years) in the healthy controls.

Furthermore, the repeatability of the measurement of the transparency in the middle layer of the cornea was better than that in the posterior and anterior layers of the cornea. These findings are in accord with the results of previous (intra-day) studies on cohorts of subjects with keratoconus^17,23^, and among healthy subjects^6,17,18,23^. However, to the best of our knowledge, no studies have been carried out to determine the inter-day repeatability of corneal densitometry measurements and thus, the results from this investigation cannot be compared. The better repeatability of measurements in the middle layer is explained by the structure of the stroma causing negative interference, while the anterior and posterior layers are made up of the epithelium and endothelium , with the respective basal membrane layers causing increased scattering^3^. In addition, tear film quality could affect the densitometry measurements of the anterior layer. Furthermore, the ambient illumination could affect the measurements, but this aspect was considered, and was deemed to be similar on all measurement occasions. The inter-day repeatability of measurements is of considerable importance as changes between visits are evaluated in the clinical setting. Due to the lower biomechanical strength in corneae affected by keratoconus^13^, day-to-day variations of the shap cannot be excluded which will affect the repetability of the measurements. In fact, the repetability of the densitometry values presented in this investigation and the repeatability of the keratometric values presented in the previous investigation^10^ differes between Day 0 and Day 3. This suggests the importance of considering the inter-day aspects of the repeatability of the measurements in order to accurately provide cut-off limits at which a true change can be diagnosed. However, future investigations are warranted to further elucidate the inter-day effects on the repeatability of measurements.

A strength of this investigation is that both intra-day and inter-day repeatability were determined, allowing comparisons. Importantly, we provide repeatability limits for the mean of replicate measurements on each occasion and prediction limits for single measurements. In calculating the prediction limits the variability of the four measurements on each day (Day 0 and Day 3) was included in order to give more accurate prediction limits, which would otherwise have been too narrow^24^. The 95% CIs of the inter-day repeatability using a mean of replicates are, as expected, considerably narrower than the 95% CIs of the prediction limits for single measurements. If these differences are not taken into consideration when determining changes in corneal densitometry between visits, there is a risk of misinterpreting the findings.

Corneal transparency has been suggested as a useful parameter for the early detection of keratoconus^14^. However, the findings of this study strongly contradict this, as the 95% CIs of the inter-day repeatability in subjects with keratoconus and in healthy controls overlapped. Previous studies have also shown that the transparency decreases with increasing keratoconus disease severity^15^, which indicates that corneal transparency could be used in the detection of progressive keratoconus. It has also been suggested that transparency could be used as a parameter in the evaluation of the efficacy of treatment with CXL^25^. The effect of CXL on corneal transparency has been reported in several clinical studies^26,27^, and has also been used as the primary outcome variable^28^.

However, the high variability of these measurements limits their clinical use. The ICC is lower than that for other parameters commonly used in the management of keratoconus. This suggests that the variability is due, not only to inter-subject variability, but also to intra-subject variability^29^. This, per se, indicates poor repeatability. In fact, the repeatability of corneal density measurements is poorer than that of other commonly used parameters. The CV% is more suitable for comparisons of the repeatability of various variables as it is a unitless parameter. The CV% of measurements of the densitometry parameter with the best repeatability (2–6 mm, posterior layer) in subjects with keratoconus was 1.89, which is a factor of 3 higher (poorer) than that in Kmax (CV% = 0.57) from the same cohort of subjects^10^. When comparing the CV% of the densitometry parameter with the poorest repeatability (10–12 mm, anterior layer, CV% = 9.23) to the CV% in Kmax the factor is 16. The findings of this study thus show that, in its current form, densitometry cannot be used for the detection of progressive keratoconus. As there is no gold standard for the diagnosis of progression, we must use parameters with a high level of repeatability that define levels at which changes in parameter magnitude indicate progression. For similar reasons, densitometry is also unsuitable for the evaluation of the efficacy of treatment with CXL as the high variability would require an unrealistically high number of participating subjects (sample size) to yield adequate power.

Nevertheless, an objective assessment of the transparency of the cornea would be of considerable interest given its structure. Apart from the commercially available Pentacam HR Scheimpflug camera, other imaging modalities based on OCT and in vivo confocal microscopy have been shown to provide highly accurate quantitative measurements of corneal structures and their changes in various disease entities, including keratoconus^30,31^. Anterior segment OCT has been shown to provide reliable values of corneal optical density^32^. Measuring not only the backscattering of light, but also the birefringence properties of the cornea, which are also dependent on its collagen structure, could provide new indices for disease characterization. A recent study demonstrated the excellent repeatability of phase retardation measurements with polarization-sensitive OCT, and better repeatability of epithelial thickness measurements than spectral domain OCT^33^. It is possible that combining different imaging modalities that measure different properties of the cornea could increase the accuracy in disease detection and the diagnosis of progression in keratoconus^30,34^.

In summary, this study provides normative repeatability values for inter-day measurements of corneal transparency in subjects with keratoconus and in healthy controls. The use of single measurements or the mean of replicates on each occasion has been considered. The repeatability of these measurements was generally poor, but the best repeatability was found in the 0–2 and 2–6 mm zones, in the middle and posterior layers of the cornea. Nevertheless, corneal densitometry is not suitable for use in subjects with keratoconus due to the poor repeatability of measurements. New techniques such as anterior segment OCT and possibly polarization-sensitive OCT could provide variables with high accuracy and improved repeatability allowing for use in clinical practice.

## Methods

This investigation is a post-hoc analysis of data from a prior publication^10^.

The study was conducted at the Department of Ophthalmology at Skåne University Hospital, Lund, Sweden, according to the declaration of Helsinki. The Regional Ethics Committee in Lund, Sweden, approved the studies (No. 2015/373).

### Subjects

Patients with keratoconus (n = 25) fulfilling the inclusion criteria described below were consecutively enrolled after signing an informed consent form. The inclusion criteria were: keratoconus Stage ≤ 2, with no history of, and no current signs of, other ocular pathology, including ocular surface disease and external diseases such as dry eyes and atopy. Only subjects who had not undergone prior ocular surgery and who were aged ≥ 18 years were recruited. Pregnant and breastfeeding women were also excluded. Contact lens wear was discontinued at least 2 weeks before the measurements were made. Keratoconus was diagnosed clinically and by examination using the Pentacam HR, a Scheimpflug-based tomographic system (Pentacam HR, version 1.20r10, Oculus Optikgeräte GmbH, Wetzlar, Germany). The technical features of this system have been described elsewhere^35^. The default setting of 25 images/s was used. The sagittal curvature pattern, posterior and anterior elevation maps, and corneal thickness pattern were assessed, and information from the Belin-Ambrosio Enhanced Ectasia Display was used in the diagnosis of keratoconus^36^.

Healthy controls (n = 25) were enrolled from among medical students and residents in ophthalmology after signing an informed consent form. The inclusion criteria were: age ≥ 18 years, no history of any ocular pathology or previous ocular surgery. Pregnant and breastfeeding women were excluded. Ocular pathology was excluded by clinical examination and by examination using the Pentacam HR.

The Pentacam HR measures densitometry in concentric annular segments from the centre of the cornea outwards (0–2 mm, 2–6 mm, 6–10 mm and 10–12 mm) and at different depths (layers) in these annular segments (anterior, middle, posterior, and a total value of these layers).

### Examination

Measurements were made on two separate occasions, three days apart (denoted Day 0 and Day 3). Four consecutive measurements were made on each day with the Pentacam HR by the same examiner (IG). Subjects were instructed to blink between measurements, but not to lean back. Measurements were made during normal working hours without taking corneal diurnal variation into account. Only examinations deemed “OK” by the Pentacam HR were accepted. The right eye was examined first, then the left, if both eyes were eligible for inclusion. This reflects the normal clinical setting where both eyes of the patient are usually examined. When recruitment to the study was complete, computerized randomization was performed to select one eye per subject for analysis.

### Statistical methods and calculations

The values obtained for the four replicate measurements on Day 0 and Day 3 were averaged for each day and used to calculate the inter-day repeatability for the clinical scenario when using the mean value of measurements to assess progression of keratoconus. When calculating prediction limits in the clinical scenario when single measurements are used to assess progression, the variance between replicate measurements was included in the calculation to provide more accurate results.

IBM SPSS Statistics 22 Windows (IBM Corporation, Armonk, NY, USA) and SAS Enterprise Guide 6.1 for Windows (SAS Institute Inc., Cary, NC, USA) were used for statistical analyses. A p-value below 0.05 was considered significant. Descriptive statistics are given as subject mean, standard deviation (SD), and minimum and maximum values. Repeatability was assessed by calculating the within-subject standard deviation, precision, repeatability coefficient, intra-class correlation and coefficient of variation with associated confidence intervals (CIs)^19,20,29^. Kendall’s tau was used to assess the relationship between the mean and SD, and natural logarithm transformed data were analysed when appropriate. The limits of agreement were calculated using the replicates and a linear mixed-effect model (denoted prediction limits)^24^. A professional medical statistician was consulted.

### Definitions and abbreviations

- Within-subject standard deviation (S_w_). The square root of the variance between subjects.
- Precision = 1.96 × S_w_. The difference between a measurement and the true value should lie below this limit in 95% of the measurements.
- Repeatability coefficient (RC) = 2.77 × S_w_. The difference between two measurements should lie below this limit for 95% pairs of observations.
- Coefficient of variation (CV). S_w_ divided by the total subject mean.
- Intraclass correlation coefficient (ICC). The variance between subjects divided by the variance between subjects plus the variance within subjects.
- Prediction limits (PL) = 95% CI for differences between two future single measurements.

## Supplementary Information

Supplementary Information 1.

Supplementary Information 2.

Supplementary Information 3.

Supplementary Information 4.

Supplementary Information 5.

Supplementary Information 6.

Supplementary Information 7.

## Data Availability

All data are available as “supplementary information”.
