# Supplementary figures and images for: The intra- and inter-day repeatability of corneal densitometry measurements in subjects with keratoconus and in healthy controls
Source: Sci Rep. 2023 Apr 5;13:5566. doi: 10.1038/s41598-023-32822-y (PMC10076276; doi:10.1038/s41598-023-32822-y)

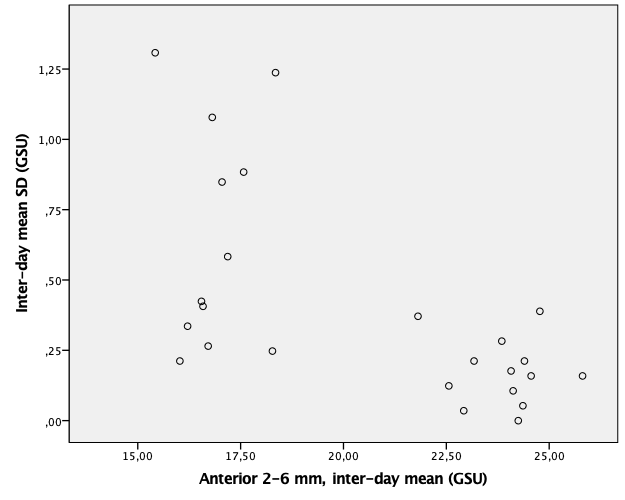

Supplement: Supplementary file 1 — Supplementary Information 1. [file 41598_2023_32822_MOESM1_ESM.png]

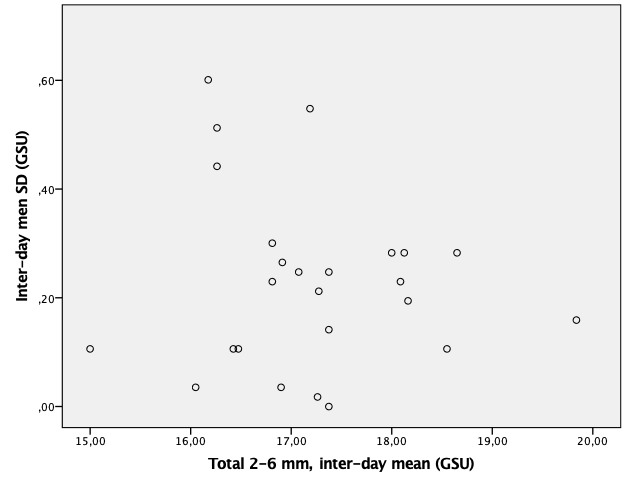

Supplement: Supplementary file 2 — Supplementary Information 2. [file 41598_2023_32822_MOESM2_ESM.png]

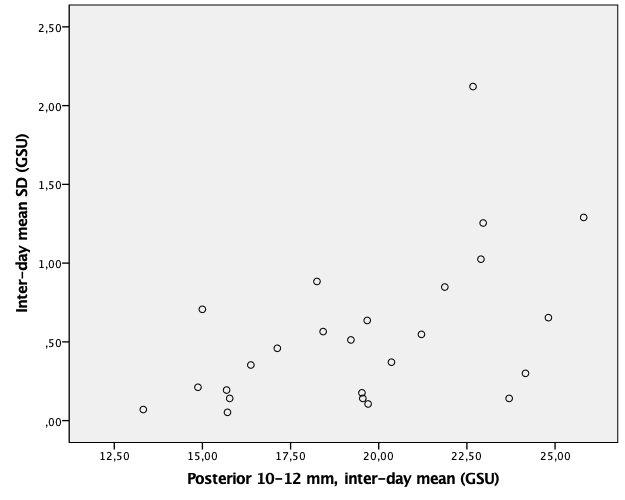

Supplement: Supplementary file 3 — Supplementary Information 3. [file 41598_2023_32822_MOESM3_ESM.png]
